# Nurses' experiences of participating in employee assistance program and factors influencing its effectiveness: a qualitative study

**DOI:** 10.3389/fpubh.2025.1614890

**Published:** 2025-08-14

**Authors:** Xuanhao Fan, Jiehao Zhuang, Zhongqing Chen, Ziyi Xiong, Niu Yang, Jiangfeng Pu, Peng Xu, Lifang Chen, Huigen Huang

**Affiliations:** ^1^School of Nursing, Guangdong Pharmaceutical University, Guangzhou, Guangdong Province, China; ^2^Department of Nursing, Guangdong Provincial People's Hospital (Guangdong Academy of Medical Sciences), Southern Medical University, Guangzhou, Guangdong Province, China

**Keywords:** effectiveness, employee assistance program, experience, factors, nurses

## Abstract

**Introduction:**

Employee assistance program (EAP) as an effective modern method for improving the physical and mental health of nurses. To understand the experiences of clinical nurses participating in the EAP, to explore the relevant factors influencing its implementing effect, to provide reference for developing a more effective and reasonable EAP in healthcare organizations, to implement targeted assistance for nurses, and to promote the physical and mental health development of nurses.

**Methods:**

A descriptive research method was used to conduct semi-structured interviews with 14 clinical nurses. Interviews lasted between 25–40 min. The themes of the interview data were distilled through thematic analysis using NVivo 15 software. Sample size was subject to content saturation.

**Results:**

The mean age of the participants was 36.29 years, and the mean duration of clinical work was 14.07 years from different departments. Two themes and six sub-themes were identified for nurses' experience of participation in EAP. Seven themes and 15 sub-themes were identified as factors affecting the effectiveness of EAP implementation from the government, hospital, EAP, and nurse level.

**Discussion:**

This study innovatively identified nurses' experience of participating in the EAP and the factors influencing its effectiveness obtained from a qualitative analysis perspective. Nurses could have a variety of positive experiences by participating in an EAP, with improved mental health status being the main focus. However, the long-term and professional nature of EAP was still deficient. This study found that there were a variety of factors affecting the effectiveness of EAP implementation, including the government's financial support, institutional safeguards, and the participation and support of hospital management, which were the prerequisites for the effectiveness of the program. The integration of multiculturalism and the enhancement of publicity could promote the two-way understanding between nurses and EAP, while ensuring that the EAP had long-term, flexible, targeted, and professional characteristics was key to its effective implementation. Therefore, in future studies, there remains a need for the government to establish universal team training and implementation programs, while hospitals must personalize applications and maintain sustained implementation to collect nurse feedback, thereby optimizing the EAP and safeguarding the occupational health of nurses.

## 1 Introduction

As global population aging intensified and chronic diseases and mental health issues became more prominent (1), the demand for nursing services increased, placing stricter demands on the quality of care. However, the global shortage of healthcare workers remained a serious concern. In 2020, the World Health Organization (WHO) reported a global shortage of 15 million healthcare workers, and it was expected that by 2030 there would be a global shortage of up to 18 million healthcare workers, with the shortage of nurses and midwives reaching 5.7 million (2). By the end of 2023, China had 4.00 registered nurses per 1,000 population (3), which was still below the WHO proposed median global density (4.86 registered nurses per 1,000 population) (4). The shortage of nurses led to an increase in nursing workload, and out of responsibility for the need to provide quality nursing services, would face greater pressure, which in turn made nurses prone to physical and mental health problems, and in serious cases led to nurses producing turnover behavior (5), further increasing the work pressure of clinical nurses, forming a vicious circle.

In addition to alleviating the nursing manpower shortage, improving the physical and mental health status of nurses working in clinical settings is equally crucial in addressing the vicious cycle. It has been found that there were various interventions to improve the mental health of nurses, such as mindfulness stress reduction therapy, cognitive behavioral therapy, and emotional release therapy, as well as the development of stress management plan (5). Similarly, there were physical activity and healthy eating programs aimed at improving physical health (6), workplace intervention programs to alleviate musculoskeletal pain in nurses (7), and even a study found that providing nurses with music therapy reduces levels of stress-related biomarkers (8). Many researchers had explored diverse approaches to enhance nurses' physical and mental health, with promising results. As a result, comprehensive programs integrating these effective strategies were increasingly adopted by healthcare organizations.

In recent years, Employee Assistance Program (EAP) had been introduced as a modern and effective tool to provide appropriate assistance services to the management of healthcare workers (9, 10). EAP was a set of systematic, long-term welfare programs provided by an organization for its employees, through which professionals provided employees with diagnosis, assessment, training, professional guidance and counseling to help them solve various psychological and physiological problems timely (11). Studies had shown that EAP provided healthcare workers (including nurses) with a wide range of assistance to improve their physical and mental health, reduce workplace stress, and bring positive results to both the individual employee and the hospital as a whole (12, 13). EAP functioned as a benefit program that provided comprehensive support for nurses. In addition to enhancing their physical and mental health status, the program addressed work-related conditions and quality of life issues. It helped nurses improve their wellbeing at work which was a personal positive work condition (14, 15). This positive work condition reduced nurses' turnover intention. It was evident that nurses' self-improvement and enhancement from the EAP was a positive effectiveness provided by the EAP. Thus, in order to further enhance the positive effects of EAP for nurses, the relevant factors on the effectiveness caused by the process of EAP implementation needed to be analyzed in depth, but the effectiveness of nurses' participation in EAP was influenced by a variety of factors, the complexity of which needed to be further explored to avoid too many confounding factors affecting the positive effect. Currently, more studies had validated the effectiveness of nurses' participation in EAP through quantitative methods using standardized measurement tools, and had explored the effectiveness of the content, form of implementation, and duration of EAP on nurses (16). While numerous quantitative studies demonstrated that EAP yielded positive outcomes for nurses, most focused primarily on individual health and behavioral indicators, such as anxiety, depression, and stress. The analysis of effectiveness from the nurses' perspective had been dominated. But these studies failed to explore nurses' experiences during EAP participation or systematically examine the factors influencing EAP effectiveness throughout the entire intervention process. Additionally, due to the absence of a standardized EAP evaluation index system tailored for nurses, as well as a lack of tools to quantify experiential and effectiveness, analyzing complex factors through quantitative studies was difficult. Qualitative research could emphasize the experience of subjectivity and also helped to fully analyze the EAP implementation process (17). Thus, it could demonstrate nurses' participation experiences, identify potential factors affecting EAP's effectiveness, and provided insights for developing a comprehensive, systematic EAP evaluation index system (18). However, there were still fewer qualitative studies that analyzed the experience of nurses' participation in EAP and the factors influencing its effectiveness. Obtaining feedback from the perspective of clinical nurses and analyzing the hinder factors of the implementation of EAP throughout the entire process are conducive to timely adjustments of the EAP, reducing the cost of its implementation, and improving the effects of nurses' participation. From the perspective of the job demands-resources (JD-R) model (19), nursing staff shortages, heightened nursing quality requirements, and increased work stress negatively impacted nurses' physical and mental health through the health impairment pathway, while the EAP, as a work resource, enhanced nurses' wellbeing via the motivational pathway. Simultaneously, an interaction was observed between job demands and resources, with an effective EAP being the key to reducing work demands. Therefore, based on the perspectives of clinical nurses who have experience in EAP participation, this study deeply explores the experience of nurses' participation in EAP and the factors influencing its effectiveness. This study can provide reference value for the development and improvement of EAP in other healthcare institutions, which can help to form an EAP with individualization and provide effective assistance to nurses.

## 2 Research objectives

This qualitative study explored nurses' experiences of participating in the EAP and the factors influencing its effectiveness. Therefore, we proposed the following research questions for this study: (1) What experiences did nurses gain from participating in the EAP? (2) What factors influenced the effectiveness that nurses gained from participating in the EAP? We recruited two eligible nurses for pre-interviews through the developed inclusion criteria, and revised the final interview outline based on the feedback from the pre-interviews, as shown in Table 1.

**Table 1:** Interview outline.

**Question**
1 Do you know anything about Employee Assistance Programs (EAP)? Tell us about your understanding
2 What is your goal for participation in the EAP?
3 What did the program carry out when you participated in the EAP? What professionals have assisted you?
4 What effects did you get from participating in the EAP? What could have an impact on the effect? You can talk about it in the short and long term
5 What kind of advice would you give to the EAP?

## 3 Materials and methods

### 3.1 Participant recruitment

From November 2024 to February 2025, clinical registered nurses in a tertiary hospital in Guangdong province who had participated in the hospital's EAP. This EAP was implemented over a period of 6 months and focused on mental health management. The program was implemented in three sequential phases: (1) An anonymous online assessment of nurses' mental health needs, work environment challenges, and improvement suggestions regarding psychological and occupational stressors; (2) Mental health promotion activities delivered through both online and offline formats; and (3) Individualized mental health counseling sessions provided on a one-to-one basis. The topics of the second phase of the program included member awareness, identification and improvement of depressive mood, relief of work stress, reduction of anxiety, enhancement of nurses' professional identity and nurses' positive relationships, enhancement of nurses' life satisfaction, work wellbeing, and mental health management seminars. Outcome was the change in depression and wellbeing scores after the nurses received the EAP intervention. Because it was a randomized controlled trial study, a total of 88 nurses participated, 43 of whom served as the control group (15) were selected by purpose sampling method as interview subjects. Inclusion criteria: (a) clinical registered nurses who had participated in the EAP; (b) no clearly diagnosed serious physical or mental health condition; (c) informed consent and voluntary participation in this study. To ensure a diverse range of interview subjects, this study recruited subjects from various departments, including internal medicine, surgery, emergency medicine, intensive care unit (ICU), operating rooms, and obstetrics and gynecology (OB-GYN). The selection criteria accounted for both novice and experienced nurses. The sample size was determined by information saturation (20). Thematic coding was achieved after interviewing 12 participants. Subsequent interviews with two additional participants confirmed that no new themes emerged, as the data consistently reinforced the existing thematic coding (21). Therefore, it was posited that the content of interviews 13 and 14 could be interpreted by the themes that had been coded a priori. This was considered saturation of the interview data and the data collection was stopped.

### 3.2 Data collection method

In following the systematic process, the interview outline was developed based on the purpose of the study with a systematic literature review (22). Developing a preliminary interview outline based on the research theme and further refining it through group discussion. Two eligible participants were recruited for pre-interviews prior to the formal interviews, and the information was included in subsequent analysis because the outlines required fewer revisions and the information from the pre-interviews was consistent with the theme of the study. Qualitative research required semi-structured, face-to-face, in-depth interviews with participants. Before each interview, the researcher (XF) obtained informed consent from the participants and explained the purpose, methodology, and content of the interview. Interviews were conducted in the participant's department to ensure that they were carried out in a quiet environment and noted, to protect the privacy of the participants, sound recordings were only carried out after consent had been obtained, two researchers (XF and JZ) were responsible for questioning and noting, respectively, using serial numbers N1–N14 in place of real names. Sound recordings and text materials were also labeled accordingly using N1–N14. Interviews were conducted in plain language and participants were encouraged to fully express their feelings and thoughts. Active listening, clarification and probing techniques were used and non-verbal cues were noted. Each interview lasted between 25 and 40 min.

### 3.3 Data analysis methods

Data collection and analysis in this study were conducted simultaneously. The verbatim recording of the sound recordings will be completed within 24 h after each interview, and for the interviews that were not sound recorded, they will be improved interview record in time according to the researcher's memory, and the non-verbal information recorded in the interview process must also be integrated into the document, and the complete interview data will be given to the participant for feedback to ensure that the information in the interview data is the same as what the participant expressed at that time. We used thematic analysis and NVivo 15 software to complete the organization and coding of the data. Two researchers (XF and JZ) read the interviews repeatedly to complete the analysis of the data, the process that required neutrality and openness. Two researchers (XF and JZ) conducted complete coding of all interview data, with any coding disagreements resolved through discussion involving a third researcher (HH) to reach consensus. We divided each interview text into individual units of meaning and coded them. Interview data collection was synchronized with thematic coding by importing the text into NVivo, marking the sentences or paragraphs as relevant to the research questions to serve as reference points, coding sub-themes through the reference points and gathering all data relevant to each potential sub-theme, finally categorizing the sub-themes to form themes (23, 24). Reflection was also ongoing throughout the study process. By summarizing the information, we gained a deeper understanding of the meanings expressed by the participants. We completed the report according to the Standards for Reporting Qualitative Research (SRQR) (25), specific checklist showed in Supplementary material.

## 4 Results

### 4.1 Sample

A total of 14 participants participated in this study, among which 10 were female and four was male, and the average age of all participants was 36.29 years old (30–54 years old). The average duration of clinical work was 14.07 years (2–34 years). The largest number of participants from Internal Medicine. Three participants refused to be sound recorded. The pre-interview results (N1 and N2) demonstrated substantial relevance to the research questions and were therefore included in the final analysis. The demographic characteristics of participants are shown in Table 2.

**Table 2:** The demographic characteristics of participants.

**ID**	**Gender**	**Age**	**Marital status**	**Education level**	**Professional title**	**Clinical working time (years)**	**Department**
N1	Female	39	Married	Bachelor	Senior	17	Internal medicine
N2	Female	32	Unmarried	Bachelor	Junior	9	ICU
N3	Female	33	Married	Bachelor	Junior	13	Internal medicine
N4	Female	30	Married	Bachelor	Junior	8	ICU
N5	Male	43	Married	Master	Intermediate	21	Internal medicine
N6	Male	39	Married	Master	Intermediate	18	Surgery
N7	Female	54	Married	Bachelor	Senior	34	Internal medicine
N8	Male	31	Married	Bachelor	Junior	8	ICU
N9	Female	35	Married	Bachelor	Junior	11	Gynecology/obstetrics
N10	Female	27	Unmarried	Master	Junior	2	Internal medicine
N11	Female	30	Unmarried	Bachelor	Intermediate	9	Gynecology/obstetrics
N12	Male	30	Married	Bachelor	Junior	5	Emergency department
N13	Female	50	Married	Bachelor	Senior	30	Operating room
N14	Female	35	Married	Bachelor	Intermediate	12	Surgery

ICU, intensive care unit.

### 4.2 Experience of participating in the EAP

#### 4.2.1 Positive experience

##### 4.2.1.1 Promotion of mental health

The majority of respondents felt that participation in the EAP enabled them to fully recognize and improve their mental health status. Several respondents in the study indicated that participation in the EAP could help them relax physically and mentally, especially after work. N9 emphasized that attending EAP at the end of the work day was not a waste of time, but rather a full use of time for relaxation, while N14 considered that such a state of relaxation was conducive to alleviating work-related stress. It could be seen that an EAP could provide nurses with a positive experience that promoted mental health with the key of stress relief. As hospital EAP were predominantly centered on mental health activities, nurses most frequently reported improvements in mental health as their core positive experience. Notably, nurses frequently experienced high-stress conditions, and their participation in the EAP's diverse thematic activities effectively reduced stress levels while generating multidimensional positive experiences.

##### 4.2.1.2 Improvement of interpersonal relationships

Participating in the EAP helped to improve interpersonal communication skills, which improved one's own communication ability and thus improved interpersonal relationships. N9 emphasized that her characteristic was straightforward, and she was prone to cause verbal harm to others when communicating with them, which could lead to tension in interpersonal relationships, and that mastering the communication skills after participating in the EAP could effectively improve interpersonal relationships. In addition, mastering communication skills could reduce work pressure and improve interpersonal relationships. N12 considered that in the face of an increasingly busy and stressful clinical work environment, mastering interpersonal communication skills and being able to communicate appropriately with patients, their families, and colleagues was conducive to reducing work pressure, especially in emergency departments, intensive care units, and other departments that required communication with critically ill patients and their families. The reduction of work pressure was conducive to nurses being more patient, providing more meticulous patient care, communicating more appropriately with patients' families, cooperating more tacitly with colleagues to complete the work and forming harmonious interpersonal relationships. By promoting interpersonal harmony, these relationships not only enhanced coworker collaboration but also served as an ongoing motivator for nurses.

##### 4.2.1.3 Mastering mental health management skills

When confronted with abnormal psychological states, nurses needed to take timely measures to cope with them. In addition, nurses were also needed to recognize them in time for early prevention, so it was meaningful to acquire mental health management skills. Respondents considered that participating in EAP could help them learn about mental health, and at the same time, they could master mental health management skills, so that they could accomplish timely intervention before abnormal states might occur, and could obtain better results. Therefore, acquiring mental health management skills could bring long-term and positive effects to nurses. Although the EAP did not provide formal training in mental health management, nurses' participation in diverse mental health activities fostered greater awareness of mental health through personal reflection and peer interaction, ultimately cultivating proactive self-manage strategies.

##### 4.2.1.4 Facilitating career development

Each nurse's career was different, and how to help their career development was an issue that each nurse needed to consider, which was related to the nurse's work remuneration, social contribution and so on. Respondents said that when participating in the EAP, they could gain experience through communication with others, especially when participating in seminars, where nurses could share their work experience and discuss their career plans with each other. Learning from others' successful work experience and career planning through dialectical learning could help nurses' career development.

#### 4.2.2 Negative experience

##### 4.2.2.1 Insufficient long-term

EAP had a short duration and nurses might have difficulty maintaining long-term effects. N6 emphasized that nurses were busy with their clinical work and needed to receive a lot of information every day, and in this case (15), the EAP was conducted for only a short period of time and lacked long-term “stimulation,” which made it easy for nurses to forget the experience of participating in the EAP. Other respondents also indicated that the EAP they had participated in had been in place for a relatively short period of time, and they did not believe that the EAP would have a long-term impact on the nurses. Nurses faced stressful work situations for long periods of time, which increased their need for long-term assistance. Short-term assistance only helped nurses for a short period of time, and EAP that lacked sustainability did not provide long-term benefits for nurses, affecting the retention of positive experience.

##### 4.2.2.2 Insufficient professionalism

The EAP had to be professional, mainly in the sense that the EAP team had a strong professionalism and provided professional assistance. Respondents expressed concern that the EAP still needed to further strengthen its professionalism because its professionalism had not met the expectations in their minds, including the form and content of its implementation, and that it was even possible for hospital to collaborate with an external EAP professional team in order to provide more professional assistance to nurses. In China, EAP conducted in hospitals were still based on an internal model, with teams composed of personnel from the hospital itself. Although the demand for nurses was clearer, the lack of professional training and the absence of a complete training program for EAP professionals by either the government or the hospitals had resulted in professionalism issues that still affected the nurses' experience.

### 4.3 Factors influencing the effectiveness of EAP

#### 4.3.1 Government level

##### 4.3.1.1 Lack of policy support

In recent years, the concern for nurses' physical and mental health problems had gradually increased, and a variety of intervention programs had been effectively developed. If the government could provide policy support based on the EAP, it helped to form institutional safeguards, and was also conducive to obtaining economic support for carrying out the program. In China, however, this was still in its infancy. Respondents said that the EAP in hospitals, as a free assistance program, had difficulty in solving the problems of manpower, materials, venues, and other resources needed to carry out the program when there was a lack of financial support, which affected the implementation of the program. When there was a lack of institutional support, there might be confusion in the conduct of the program, making it difficult for nurses to seek assistance through fast and reasonable ways. It could be seen that the lack of economic support and institutional protection would affect the normal development of EAP. An important part of ensuring the smooth implementation of EAP was providing policy support to guide the formation of a complete hospital EAP system.

#### 4.3.2 Hospital level

##### 4.3.2.1 Degree of management attention

EAP in hospitals had to be valued by management as a way to increase nurses' recognition of EAP. Management attention to the EAP was reflected in the management support and participation. When the management improved the support for EAP, it showed that the management recognized the purpose and direction of EAP, and the nurses kept in line with the management, considered that the development of EAP was necessary, and participated in EAP with a positive attitude. The implementation of EAP needed the management to actively participate in it, and communicate with the EAP team in-depth from the management's point of view, and provide reasonable suggestions to promote the smooth implementation of EAP.

##### 4.3.2.2 Unique EAP culture atmosphere

Different hospitals had unique hospital culture, which contained the core values of the hospital and established the goals and behaviors of healthcare workers, thus shaping the cultural atmosphere of the hospital. Combining the EAP with the cultural atmosphere could result in assistance services that were compatible with the hospital. Respondents believed that the lack of EAP combined with the hospital culture was not conducive to the development of EAP, and that a unique EAP needed to be constructed, and that EAP could even be combined with the hospital culture to form a unique Intellectual Property (IP), which helped to promote the application of EAP. If the combination of EAP and hospital culture could be understood as a “macro” combination, then the further combination of EAP with departmental culture was a “micro” combination, creating a unique EAP cultural atmosphere in the department, which was conducive to the participation of nurses and obtaining positive results. Therefore, combining the culture of the hospital and department was the precise optimization of the EAP. The hospital formed a standard strategy to carry out the EAP, and then the department modified and applied it with its cultural characteristics, which was conducive to the active participation of nurses in the activities, and at the same time, it was beneficial for the application of the EAP with different healthcare workers.

#### 4.3.3 EAP level

##### 4.3.3.1 Existing problems with EAP

Deficiencies in the model, implementation format, and content often affect the effectiveness of EAP, and in particular, problems with the EAP implementation process often act as barriers to positive outcomes for nurses. This study summarized three existing problems of EAP as perceived by the respondents, including insufficient publicity of the program, inadequate understand of nurses, and manpower deployment problems. Respondents considered that the EAP was not sufficiently publicized in the hospital, which resulted in nurses not knowing exactly what they were participating in before engaging in the activities. Reasonable publicity facilitated nurses' active engagement with EAP activities while enabling informed participation decisions. This approach ensured that nurses in need could access appropriate assistance and minimized resource inefficiencies. The EAP not known enough about the nurses, its overall plan could not be developed according to the needs of the nurses, and when the expected needs of the nurses were difficult to be met, then it could not form a positive effect. Respondents considered that this situation might be due to 5the lack of communication between the EAP and the nurses, and the lack of feedback from the nurses, as well as failure to gain the trust of the nurses. It was essential to enhance the EAP team's professional competencies, while systematically collecting nurses' feedback and modification suggestions regarding the program. EAP development required adequate manpower allocation. N1 indicated that he felt that there was a shortage of team members when he was involved in the EAP, and N13 felt that a large number of volunteers needed to be recruited to ensure that the EAP was carried out. Implementation of the EAP tended to be hampered by the inability to optimize the allocation of staff to provide assistance services.

##### 4.3.3.2 Characteristics required for EAP

When EAP needed to be carried out effectively and rationally, the important characteristics of EAP needed to be fully considered. Respondents of this study proposed the long-term, flexibility, focus, and professionalism of the EAP as characteristics that influenced the effectiveness of EAP implementation. When EAP design lacked long-term which easy to make nurses forget the experience of participating in the EAP, and gradually dilute the effect of the EAP, if the EAP could maintain its long-term, which was conducive to the nurses to bring long-term positive effects. EAP needed to be flexible, such as flexibility in the timing and frequency of activities, to be able to increase the participation of nurses and to be able to respond to emergencies as they arose. When EAP design lacked focus, it was difficult to meet the needs of nurses. Although each nurse as an independent individual would have different needs for the EAP, and it was difficult to carry out targeted assistance to the individual, through in-depth investigation on nurses, the needs of nurses in different departments could be summarized, and targeted assistance could be given, which could also bring positive results. EAP needed to increase its professionalism, including a more professional team and assistance content, with respondents considering that a more specialized team could provide a more effective content of assistance, and that a lack of professionalism would have a direct impact on the effectiveness of implementation. Therefore, when important characteristics of EAP could not be valued, it was detrimental to both the implementation and optimization of EAP.

#### 4.3.4 Nurse level

##### 4.3.4.1 Personal time conflict

Time conflicts affect the degree of nurses' active participation in the EAP. As the working time of clinical nurses are non-fixed, this leads to the time when some of the activities are carried out to form a conflict with the nurses' working hours, which reduces the nurses' participation. Other respondents pointed out that the time when the EAP carries out activities may occur after the nurses have finished their night shifts, when the nurses need to get sufficient rest and do not have the energy to continue to participate in the activities, which leads to a decrease in the degree of participation. When nurses with mental health needs were required to participate in EAP activities during their post-night shift recovery periods, instead, it could have a more serious impact on their mental health. Therefore, the EAP team was required to complete the scheduling of activities based on the nurse's reality as much as possible.

##### 4.3.4.2 Perceptions and privacy concerns in the EAP

Nurses' understanding of EAP and protection of personal privacy were the influencing factors of effectiveness. More than half of the respondents said that they only had a preliminary understanding when participating in EAP, but did not explore the concept, meaning and content of EAP. Respondents hoped that they could fully recognize and understand EAP with the help of the EAP team, which would improve the effect of participation. Nurses' understanding of the EAP was partially influenced by both the program's publicity efforts and receiving preliminary orientation about its fundamental concepts and contents prior to implementation. Therefore, the EAP improved planning for program dissemination and content design. The protection of personal privacy was the basic right of nurses participating in EAP, and it was also an issue that needed to be considered from the beginning to the end of EAP, when the information of nurses was leaked, it affected the nurses' trust in EAP, and might even cause harm to nurses. N7 emphasized that when the privacy of nurses participating in EAP was not protected, it affected the implementation of the program, and might even interrupt the program, which would have a negative impact on the nurses.

**Figure 1:**
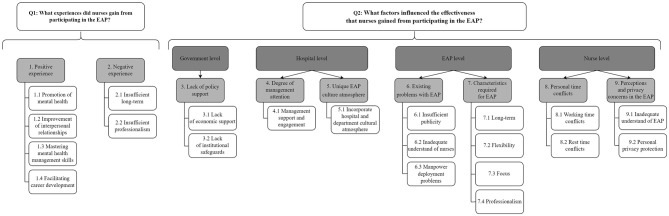
Thematic map.

The first-level themes summarized through NVivo were nine and second-level themes were 21, with a total of 131 reference points present. The specific first-level themes, second-level themes, and quotes of the above results were summarized in Tables 3, 4. Specific thematic maps were shown in Figure 1.

**Table 3:** Experience of participating in the EAP.

**First-level themes**	**Second-level themes**	**Quote**
Positive experience	Promotion of mental health	N8: “I felt very relaxed after the event, including the next few days at work, the whole person is also in a more relaxed state, which should be regarded as the body, the spirit can be relaxed in a short period of time.” N9: “Going to these programs after work will make you feel like you won't be wasting your time, but rather that you're using that time to relax.” N12: “I think the main focus when attending the EAP is mental health guidance… Have a better understanding of mental health aspects.” N14: “Because it's a mental health management program… I remember that I was quite relaxed after attending the program and was able to meet the expectation of relieving stress at work.”
	Improvement of interpersonal relationships	N4: “There are also interpersonal communication skills that are learned, including how to communicate with coworkers and patients.” N9: “Improved my interpersonal communication skills, because I am a rather blunt person, my speech will be more direct, and sometimes what I say will be hurtful to other people, which brings me tension in interpersonal relationships, after joining (EAP) I can feel that I have made adjustments, how to effectively and reasonably communicate with others.” N12: “Participation in the EAP improves communication skills to some extent. Being able to communicate appropriately with patients and families is much less stressful (at work).”
	Mastering mental health management skills	N4: “I am able to learn knowledge and skills related to mental health interventions and improve my empathy skills.” N9: “EAP provides mental health management services for our employees and we can learn a lot of useful knowledge ourselves… When mental health problems do arise at work in the future, we will be able to apply this knowledge and skills.” N10: “I understand and learn about various aspects of mental health and learn to self-prevent.”
	Facilitating career development	N11: “EAP services have benefited me… Providing me with a lot of work experience.” N12: “Participation in panel discussions where people learn and share with each other and also discuss career planning aspects (content).”
Negative experience	Insufficient long-term	N6: “However, as we are so busy in our clinical work, if the EAP program cannot be carried out for a long period of time, it will not be able to give us a long-term “stimulus,” and we will easily forget about it!” N11: “Because this EAP program of ours is not long-term, a lot of things and contents are still not quite remembered, and we may need to be reminded to have a little impression like this.” N14: “I don't think there are any long-term effects because if you can't participate for a long time, the effects aren't significant.”
	Insufficient professionalism	N6: “In my opinion, there may still be a lack of professionalism in EAP in hospitals… How about a more professional format and content to help nurses, that's what I'm looking forward to.”

EAP: employee assistance program.

**Table 4:** Factors influencing the effectiveness of EAP.

**First-level themes**	**Second-level themes**	**Quote**
**Government level**		
Lack of policy support	Lack of economic support	N5: “After all, the EAP is a free program, and the lack of economic support can change the circumstances of our program schedule, thus affecting its effectiveness.” N6: “I think it is definitely necessary to have the support of human, financial and material resources to carry out this kind of program, otherwise it is difficult to promote and apply EAP.”
	Lack of institutional safeguards	N1: “Institutional safeguards are needed… Well-established institutional safeguards allow nurses to find quick and reasonable avenues to apply for assistance.”
**Hospital level**		
Degree of management attention	Management support and engagement	N5: “EAP needs to communicate fully and effectively with the hospital's management, and the content of the program must be realized in the early stage… You need to know the hospital management's philosophy and ideas about the program, and discuss them to come up with a consistent direction.” N7: “It needs to be supported by the hospital organization, and the purpose and direction of the EAP and the leadership need to be aligned for the (EAP) to run smoothly. Management needs to give management advice on EAP program development... EAP can be better implemented.” N10: “If none of the managers support the program, then we may hold the same idea that participation in the EAP is unnecessary.”
Unique EAP culture atmosphere	Incorporate hospital and department cultural atmosphere	N5: “We need to incorporate hospital culture and design assistance programs that are unique to our hospital.” N7: “Hospitals need to improve the culture related to EAP, the lack of which, I think, makes it difficult for EAP to accomplish publicity and is equivalent to not having our own EAP.” N8: “While the hospital culture is a “general direction,” the department culture is more familiar to your department, developing a good working environment.” N12: “If the EAP is combined with an IP like hospital culture, I think it's more promotional.”
**EAP level**		
Existing problems with EAP	Insufficient publicity	N5: “Lack of effective publicity, did not reach the heart of nurses, nurses do not know what (EAP) is, affecting their feelings and understanding of the activities.” N8: “How to publicize the EAP well is necessary, otherwise it is not very effective for us to attend without knowing what is going on.”
	Inadequate understand of nurses	N10: “I feel there is a lack of feedback and trust with the EAP, and it should work better if the EAP knows what we need and makes content adjustments.” N14: “I wish the EAP would have a good conversation with our nurses or go to the unit and communicate with the nurses, then to get what help the nurses need right now, that would be effective.”
	Manpower deployment problems	N1: “Many times (EAP) lacks manpower support, and the lack of manpower makes it difficult to ensure program implementation.” N13: “The EAP needs a lot of volunteer help to form a full team.”
Characteristics required for EAP	Long-term Flexibility	N4: “If the EAP is running for a long time and I am able to participate for a long time, it can be a great enhancement for me.” N10: “I think the EAP is a long-term program, and getting services over a long period of time is the best way to get the best results, relieve stress, and maintain a healthy mindset over the long term.” N4: “Is it possible for the EAP team to be flexible about activities? For example, some activities can be done in batches or more frequently.” N5: “Through communication with nurses who have participated in the EAP, such activities were found to be beneficial, and some even requested to increase the number and duration of the activities.”
	Focus Professionalism	N6: “I think there is a screening that needs to be done before an EAP is conducted, which is to screen the population… Then the EAP can be targeted.” N10: “Hope (EAP) carries out assistance services centered on healthcare staff's requests.” N13: “It feels like nurses often “go to” the EAP for assistance, and whether the EAP team can go to different departments to conduct research and provide targeted assistance.” N6: “How hospitals can help their employees in a more professional way, including form and content.” N7: “When providing counseling services to nurses… There is still a need to find appropriate and specialized professionals who can provide targeted services to give assistance.”
**Nurse level**		
Personal time conflicts	Working time conflicts Rest time conflicts	N3: “There were a few activities that I did not attend due to scheduling conflicts. Other than that, I cannot think of any other factors that influenced my participation in the EAP.” N9: “Sometimes it's not possible to attend the EAP because of conflicting work schedules... Purposely taking time off or transferring shifts to attend (EAP) is not something I would do.” N10: “Sometimes attending the EAP coincides with the end of the night shift, and if you use the time off from the night shift to attend the program, I don't think it's effective, and you might as well save the time to go to sleep.”
Perceptions and privacy concerns in the EAP	Inadequate understand of EAP	N7: “If the nurses are participating in the EAP, they are approaching it with a resistant response (not understanding the EAP), which surely leads to negative outcomes.”N8: “I was under the impression that there was no introduction (to the EAP) and that when attending the EAP we need to be made fully aware of the EAP by the organizers, which will definitely have a positive effect.”
	Personal privacy protection	N7: “I have always emphasized that when it comes to personal privacy, there is an impact on EAP implementation and even the possibility of interruption of intervention, and being able to do a good job of protecting privacy can have a positive impact on EAP implementation.” N10: “We lack trust in the program in case it will have an impact on our career when we expose too much of our privacy to this program.”

EAP: employee assistance program.

## 5 Discussion

This study explored the experiences gained by nurses when participating in EAP from the perspective of clinical nurses. The positive experiences of nurses' participation in EAP included four aspects: promotion of mental health, improvement of interpersonal relationships, mastering mental health management skills, and facilitating career development, which mainly reflected the assistance and competence enhancement that individual nurses received from EAP. This also reflected the individual's authentic experience of self-improvement through the motivational pathway of acquiring job resources.

EAP provided a variety of assistance, and assistance related to mental health was one of the key components of the EAP (12, 26). In the face of increasing work pressure, nurses needed to have their stress relieved in order to improve their mental health status, and several studies demonstrated that participation in the EAP was effective in relieving stress and improving nurses' ability to cope with stress (27, 28). Although the results of this study focused on relieving work stress and thus gaining experiences that promoted mental health, the state of nurses' mental health had also been associated with multiple factors (13, 29, 30). Thus, a comprehensive and rational EAP design is key to making the EAP work on multiple factors so that nurses can have a positive experience in multiple ways that promote mental health.

The results of this study showed that nurses' participation in the EAP process improved communication skills, improved nurses' interpersonal relationships through good communication with patients, families, and coworkers, and ultimately formed a harmonious atmosphere that produced a positive experience in the nursing process. Harmonious interpersonal relationships relieved work pressure and improved nursing quality. At the same time, harmonious nurse-patient relationships were conducive to nurses' in-depth understanding of patients' needs, timely help and care for patients, as well as improving patient satisfaction and respect for nurses, reducing nurse-patient conflicts, and lowering nurses' psychological burden (31, 32). In addition, harmonious coworker relationships enabled nurses to maintain close contact with other coworkers in their work and improve the quality of nursing care, and getting along well with coworkers could form a congenial departmental atmosphere, which put nurses in a positive working environment to complete their nursing work (33, 34). Therefore, how to help nurses improve their awareness of different interpersonal relationships and their communication skills was the focus of nurses' positive experience from EAP. The interview results of this study focused on the improvement of interpersonal relationships at work, but the support of harmonious family relationships was also something that the EAP needs to focus on, and the EAP provided a platform for the families of nurses to learn about the work of the nurses and to deepen their understanding of each other, which will help the nurses to gain more support. We identify this as a key research direction for optimizing the assistance content.

The nurses in this study felt that they could acquire mental health management skills during their participation in the EAP process, which brought about multiple positive experience. On the one hand, the nurses were able to consistently apply the skills to themselves, bringing positive effects in preventing their own mental health problems. On the other hand, nurses were able to apply the skills to others, such as patients, coworkers, and family members. When applying the skills to patients, especially critically ill patients, they could make them cooperate with treatment and care in a positive frame of mind and promote their recovery, and when teaching the skills to coworkers or family members, they could help to form a healthy atmosphere in the department and family.

Career development for nurses helped nurses grow as individuals, improved their salaries and gained recognition and social respect within the profession (35, 36). Providing nurses with career development assistance could lead to long-term positive experiences for nurses. It was worth noting that the nurses in this study considered that EAP helped their career development, mainly through communication and discussion with colleagues to expand their own work experience, as well as drawing on the career development plans of excellent nurses in the process of discussion to form a career development path suitable for them. Nurses globally perceive a discrepancy between their actual social status and societal expectations, with Chinese nurses facing particularly persistent stigmatization (37). It was the EAP focus on nurses' needs for social status, and practical help in planning their careers that had been the strength of the EAP.

Nurses' negative experiences of EAP participation included both lack of long-term and professionalism, which were perceived by nurses as deficiencies in the EAP.

The EAPA defined EAP as a program that needed to be carried out systematically and over a long period of time, indicating that hospitals needed to consider the importance of EAP as a long-term program (11). However, the results of this study showed that nurses perceived the EAP as lacking a long-term, which in turn lacked continuous “stimulation” and led to the forgetting of memories of participating in the EAP process, which in turn diluted positive experiences. Therefore, it is necessary to maintain the continuity of the EAP.

Nurses' experience of what they gained from participating in the EAP was related to the degree of professionalism of the EAP team, and this study found that nurses expressed concerns about the professionalism of the EAP. A professional team was a prerequisite for the development of an EAP. Currently, most EAP carried out in Chinese hospitals were based on an internal model, in which the hospitals formed their own EAP teams to provide assistance to nurses (28). Although the EAP team was more familiar with the nurses in the internal model, the lack of systematic training resulted in a lack of professionalism in conducting EAP (39). The external model was able to provide more specialized assistance by a team outside the hospital, but it was more costly, which added to the financial burden of the hospital, and at the same time was less familiar with the nurses, which made it difficult to meet the needs of the nurses. Therefore, the development trend was to form EAP teams that were specialized and able to meet the needs of nurses by mixing internal and external model (12). The EAP in which the interviewees in this study participated involved adopting an internal model. The lack of specialized training received by team members may have contributed to the unprofessional implementation of the EAP. Simultaneously, establishing a professional EAP team necessitates structured training programs. Universal training programs need to be structured with policy support. Hospitals must adapt these programs to their specific contexts to ensure practical applicability, developing more competent EAP teams.

Nurses' participation in the EAP process was affected by a variety of factors, which required us to analyze the relevant factors affecting the effectiveness of EAP from multiple perspectives, and this study explored the four levels of government, hospital, EAP, and nurses. While the varying factors across the four dimensions affected motivational pathways, they simultaneously contributed to identifying optimal intervention points, thereby enhancing the implementation effectiveness of EAP as an organizational resource.

This study showed that there was a lack of policy support for EAP (11). On the one hand, as a welfare program that provided free assistance to nurses, EAP required sufficient financial support for its continuous implementation, and the lack of financial support affected the duration and content of EAP, and even the quality of EAP, which in turn affected the effectiveness of participation. In China, the lack of financial support had led to a predominantly internal model of EAP implementation in hospitals. Although this was a low-cost investment, it did not necessarily result in high-quality assistance and substantial benefits (16, 38). However, the EAP had been shown to produce significant effectiveness for healthcare workers. They received assistance to improve their productivity, which resulted in clear financial benefits for the hospital. Therefore, we considered that stable, continuous financial support was key to successfully implementing the EAP rather than high or lacking financial support (40). On the other hand, EAP needed a reasonable institution to ensure its smooth implementation, and developing an EAP institution that met the hospital's own needs helped to set up a specific implementation plan, while a reasonable institution enabled nurses to apply for assistance in a more standardized way, and a standardized process enabled nurses to participate in EAP more actively (41). Since the epidemic, China had witnessed a progressive expansion of policies aimed at enhancing healthcare workers' physical and mental wellbeing, which provided hospitals with implementation guidelines, though a comprehensive EAP institution remained lacking (42). In contrast, “The Nurse Midwife Health Program” was a platform supported by the Australian Government to support the health and wellbeing of nurses across the country and was a free program (43). It could be seen that a huge EAP could be constructed with policy support to provide policy assistance for its development, which would help to improve the scope and quality of EAP development.

This study indicated that the degree of management attention and the unique culture atmosphere would have an impact on the effect obtained from nurses' participation in the EAP. Management attention influenced the attitude of nurses toward EAP (44). The attention of management was mainly reflected in the two aspects of support and participation. Hospital management, as the leader of the hospital, when actively providing all kinds of support for the EAP, including human, economic, material support, nurses would believe that participation in the EAP was in line with the management's goals and ideas, which would enhance the nurses' sense of identity and trust in the EAP and help to increase the effect of participation. At the same time, the management actively participated in EAP, communicated and provided suggestions for improvement, which set an example for nurses to pay attention to their own health, and nurses would be affected, so they would be willing to take the initiative to seek the assistance of the EAP, and face the EAP with a positive attitude. Chinese hospital management demonstrated insufficient attention on EAP implementation, potentially attributable to the absence of dedicated administrative departments. The ambiguous division of labor and unclear responsibility allocation for EAP management tasks progressively diminished managerial attention, consequently compromising implementation effectiveness. St. Joseph's Health Center in Canada established an EAP management committee that closely monitored program implementation, systematically collected information on EAP impacts among healthcare workers, and formulated improvement recommendations to address implementation shortcomings (45). Study demonstrated that collaboration between EAP teams and existing hospital departments enhanced implementation efficiency, even in the absence of a dedicated EAP management department (46). The formation of a unique cultural atmosphere is mainly to integrate the EAP with the cultural atmosphere of the hospitals and departments. Different hospitals have unique cultural atmosphere, and the personalized integration of EAP and hospital cultural atmosphere is equivalent to the “localization” of EAP, which is helpful for the promotion and application of EAP in hospitals, and the further integration of EAP with the cultural atmosphere of departments enables EAP to understand the health status and needs of the nurses in different departments, and form targeted assistance. For special anniversaries or events within the hospital or department, integrating their thematic elements into EAP activities proved more engaging for nurses and enhanced their participation motivation. The EAP functioned as an inclusive culture platform, with cultural integration serving as its critical component (46). EAP team members required to align their operations with nursing work culture to enhance implementation effectiveness. Therefore, hospitals must pay attention to the formation of EAP cultural atmosphere.

The existing problems of EAP in the process of implementation directly affected the effectiveness of implementation, which mainly included three aspects, such as insufficient publicity, inadequate understanding of nurses, and manpower deployment problems. The lack of publicity of EAP in hospitals led to difficulties in the promotion and application of EAP, lack of recognition by nurses, and reduced participation of nurses, thus affecting the effect. It was necessary to emphasize the publicity value of EAP and use effective publicity methods (47). A focus on EAP dissemination also promoted nurses' understanding of the EAP. From the point of inadequate understand of nurses, there was a lack of communication between the EAP team and the nurses, and the nurses were unable to provide timely feedback, which in turn led to the EAP not accurately identifying the needs of the nurses. It was demonstrated that EAP helped to improve understanding of nurses by evaluating effectiveness in diverse ways and obtaining timely feedback from nurses (48). Consequently, we considered that EAP implementation required a nurse-centered approach. Given the absence of standardized effectiveness evaluation tools, program assessments needed to incorporate multidimensional indicators, including mental health status, participation rates, and satisfaction levels, to holistically evaluate implementation efficacy and better comprehend nurses' needs. From the point of manpower deployment problems, providing assistance to the whole hospital of nurses, covering a large area, and at the same time based on the internal model, the EAP team was prone to face the pressure of clinical duties and EAP work, and the work conflict would lead to the shortage of manpower, which in turn affected the development of EAP. There is still a lack of research on the issue of EAP manpower allocation in hospitals, and how the EAP team can rationally allocate manpower is an urgent issue to be addressed in the future.

This study suggested that EAP needed to be characterized by long-term, flexibility, focus, and professionalism. Nurses considered that EAP needed to be sustained over the long term, contributing to its long-term positive outcomes. Related studies had also considered that long-term persistence was one of the key predictors of the success of EAP in hospitals (49). Providing nurses with accurate services as needed in the long-term, and at the same time, through continuous follow-up, exploring the effects obtained by nurses participating in EAP for different time periods, and making adjustments to the overall EAP implementation planning based on the feedback information, so as to improve the benefits of EAP implementation. The long-term implementation of the EAP permitted systematic tracking of nurses' mental health status and participation motivation trends. This longitudinal monitoring enabled the team to precisely detect abnormal psychological fluctuations among nurses and reliably evaluate whether their mental conditions were deteriorating or improving. Nurses considered that the EAP needed to have flexibility in its implementation, such as an increase in the number of activities and flexible adjustment of time. Because clinical nurses faced the problem of irregular working hours, EAP could flexibly arrange the activities according to the nurses' time and increase the nurses' participation, providing options for nurses. Relevant studies had also shown that effective EAP implementation needed to be flexible (50), such as during the epidemic, when offline activities could not be carried out in the face of strict control, assistance could be provided flexibly to hospital staff through online, so that the EAP could still be carried out effectively (13). Therefore, maintaining the flexibility of EAP improved the participation of nurses and met their individual needs, which could enhance the effectiveness of EAP implementation. EAP needed to accurately identify the needs of nurses and provide targeted assistance (51). This study indicated that EAP needs to conduct in-depth surveys of nurses in different departments to fully understand the needs of nurses and provide targeted assistance, and it also needs to conduct dynamic screening of the physical and mental health of participating nurses and personalize the adjustment of assistance content. Ketelaar's assistance program screens nurses for mental health status, which in turn provides different content of assistance, reduces the cost of EAP investment, and helps nurses achieve targeted results (52). Notably, not all nurses who participating in the EAP had serious mental health issues or psychological distress (46). The EAP required establishment of an effective screening system to deliver appropriate psychological support for nurses experiencing general distress, while providing specialized referrals for those displaying abnormal mental health indicators. This tiered approach corresponded with prevailing EAP developmental trends and improved intervention precision. The professionalism of EAP mainly referred to the establishment of a professional EAP team, whose members had professional training, in order to implement EAP in a more professional and scientific way, to practically solve all kinds of nurses' difficulties, and to create a foundation for positive participation effects (42, 53). Although the gradual promotion and application of the mixed model (combination of internal and external model) could improve the professionalism of EAP teams, the formation of a standardized EAP team training program was still a future development objective.

From the nurses' personal perspective, the relevant factors mainly included personal time conflicts, perceptions and privacy concerns in the EAP. In terms of personal time conflicts, this study indicated that nurses' scheduling situation tended to affect their participation in the EAP. At present, the shortage of nurses was a serious problem. The workload of nurses had increased, so they were prone to conflict between their working hours and their activity time, which led to their low participation in the EAP. The scheduling of certain activities during nurses' post-night shift rest periods similarly diminished their participation willingness. Individual time conflict was correlated with the flexibility of the EAP, and although time conflict was difficult to avoid, how to maintain the flexibility of the EAP to mitigate the impact caused by time conflict needed to be further explored. In terms of perceptions and privacy concerns in the EAP, the main issues were nurses' lack of understanding of the EAP and personal privacy protection. This study suggested that when nurses lacked understanding of the EAP, they might respond to the EAP with a negative attitude, which might affect the implementation. Therefore, there was a need for nurses to emphasize the overall learning of EAP in the process of EAP implementation and to promote nurses' understanding of EAP (41). Personal privacy protection had always been an issue that needed to be emphasized in EAP (51, 54, 55), and if nurses' personal privacy could not be adequately protected, it was easy to cause the interruption of EAP implementation, which seriously affected the implementation effect. The stigma of mental illness frequently functioned as a barrier to EAP implementation (56), with nurses representing a professional group particularly vulnerable to such stigmatization (57). Therefore, in the process of EAP development, the rights and interests of nurses should be respected, privacy protection should be improved, stigmatization should be avoided, and nurses' trust in EAP should be improved, which would help nurses to obtain positive results.

### 5.1 Limitations

The study had several limitations. First of all, purpose sampling was used to ensure diversity in the participants, but the participants were all from the same hospital and received assistance from the same EAP, making it difficult to obtain additional information. The implementation and content of EAP vary across hospitals due to their distinct cultures, potentially limiting the generalizability of study findings. Secondly, for reasons of privacy protection, three participants were not sound recorded. Although researchers promptly supplemented the text through 24-h recall, some interview data omissions remained possible. Thirdly, the transcription and theme extraction of the interview data was initially conducted in Chinese, and there is a certain risk of translation error when translating from Chinese to English. Finally, this study lacked a research design grounded in a specific theoretical framework, potentially limiting the theoretical depth of its findings analysis.

## 6 Conclusion

This descriptive qualitative study from the perspective of clinical nurses explored the influences related to the experiences and effectiveness of nurses' participation in the EAP. Nurses could have a variety of positive experiences from the EAP, with the promotion of mental health being the most important experience. At the same time, the shortcomings of the existing EAP in terms of permanence and lack of specialization led to the formation of negative experiences. By considering the government, hospital, EAP, and nurse levels, we revealed the relevant factors affecting the effectiveness of the EAP at multiple levels and directions. The government needs to strengthen institutional support for EAP development, to provide stable economic support, to develop a standardized EAP team training program, and to provide guiding directions for EAP implementation in hospitals. Hospital management need to fully integrate the hospital culture, develop EAP for nurses in the hospital, actively interact with other hospitals to continuously improve the EAP, and in the future, EAP can cover other groups in the hospital (e.g., doctors, pharmacists, etc.). EAP's internal issues directly impacted nurses' participation results, and its multidimensional features affected long-term effectiveness and feasibility. Constructing assistance programs that are easy for nurses to access and understand based on the multidimensional features of the EAP is a focus for future research. Time conflicts, the knowledge of EAP, and privacy protection directly affected nurses' participation and indirectly influenced outcomes. Developing an EAP that is flexible and long term, removing barriers caused by time conflicts, and increasing nurses' awareness of the EAP are essential. Future research should prioritize continuous nurse feedback collection to refine assistance programs, enhance implementation quality, and generate improved outcomes for nurses.

## Data Availability

The original contributions presented in the study are included in the article/Supplementary material, further inquiries can be directed to the corresponding authors.
